# Distribution of cellular HSV-1 receptor expression in human brain

**DOI:** 10.1007/s13365-016-0504-x

**Published:** 2016-12-15

**Authors:** Richard Lathe, Juergen G. Haas

**Affiliations:** Division of Infection and Pathway Medicine, University of Edinburgh, 49 Little France Crescent, Edinburgh, EH16 4SB UK

**Keywords:** HSV-1, Brain, Hippocampus, Virus receptor, Glycoprotein D, Glycoprotein B, MAG, MYH9, PILRA, PVRL1/nectin 1, TNFRSF14/HVEM

## Abstract

Herpes simplex virus type 1 (HSV-1) is a neurotropic virus linked to a range of acute and chronic neurological disorders affecting distinct regions of the brain. Unusually, HSV-1 entry into cells requires the interaction of viral proteins glycoprotein D (gD) and glycoprotein B (gB) with distinct cellular receptor proteins. Several different gD and gB receptors have been identified, including TNFRSF14/HVEM and PVRL1/nectin 1 as gD receptors and PILRA, MAG, and MYH9 as gB receptors. We investigated the expression of these receptor molecules in different areas of the adult and developing human brain using online transcriptome databases. Whereas all HSV-1 receptors showed distinct expression patterns in different brain areas, the Allan Brain Atlas (ABA) reported increased expression of both gD and gB receptors in the hippocampus. Specifically, for *PVRL1*, *TNFRFS14*, and *MYH9*, the differential *z* scores for hippocampal expression, a measure of relative levels of increased expression, rose to 2.9, 2.9, and 2.5, respectively, comparable to the *z* score for the archetypical hippocampus-enriched mineralocorticoid receptor (*NR3C2*, *z* = 3.1). These data were confirmed at the Human Brain Transcriptome (HBT) database, but HBT data indicate that *MAG* expression is also enriched in hippocampus. The HBT database allowed the developmental pattern of expression to be investigated; we report that all HSV1 receptors markedly increase in expression levels between gestation and the postnatal/adult periods. These results suggest that differential receptor expression levels of several HSV-1 gD and gB receptors in the adult hippocampus are likely to underlie the susceptibility of this brain region to HSV-1 infection.

## Introduction

Infection with herpes simplex virus type 1 and 2 (HSV-1/2) causes orofacial, genital, cerebral, and ocular disorders. In particular, HSV-1 has been implicated in diverse acute and chronic neurological diseases including meningitis, encephalitis, and epilepsy (Gilden et al. 2007; Kleinschmidt-DeMasters and Gilden 2001).

Herpesviruses are thought to enter the brain via the olfactory pathway, as argued for HSV-1 (Mori et al. 2005) and other neurotropic viruses (Harberts et al. 2011), and then migrate to linked susceptible regions. HSV meningitis typically affects the temporal cortex, and infection of the limbic system including the hippocampus (Hpc) is implicated in HSV-1 encephalitis (Damasio and Van Hoesen 1985). Epilepsy associated with HSV-1 may also involve the Hpc because origins of focal epilepsy are typically found in the temporal lobe (reviewed in Blair 2012). An immunological study reported the presence of HSV antigen within temporal lobe and Hpc of human herpes encephalitis, with further evidence for antigen in amygdala and olfactory cortex (Esiri 1982). In mouse, inoculation of HSV into Hpc gave significantly more severe disease than inoculation into cerebellum (McFarland and Hotchin 1987). However, the tropism of HSV for the temporal brain remains unexplained.

A major factor determining species and tissue specificity of viruses is the cellular receptor(s) mediating viral entry. In contrast to most other viruses, HSV-1 entry into epithelial or neuronal cells is a complex process requiring multiple viral glycoproteins and cellular receptor molecules, including the four viral glycoproteins, glycoprotein D (gD), glycoprotein B (gB), glycoprotein H (gH), and glycoprotein L (gL), as well as the cellular receptors for at least gD and gB. Entry involves a series of concerted events based on the interaction between ligands and receptors that eventually lead to fusion of the viral and cellular membranes (Campadelli-Fiume et al. 2012a). The initial step of HSV-1 entry is low-affinity attachment of viral particles to cell-surface heparan sulfate proteoglycans (HSPGs) via gH and gB. HSPGs are very widely expressed by a broad range of different cells including neurons. This initial attachment is followed by high-affinity binding of the viral gD glycoprotein to one of the gD receptors, either poliovirus receptor-like protein 1 (PVRL1, also known as nectin 1) or tumor necrosis factor (TNF) receptor superfamily member 14 (TNFRSF14), also known as herpesvirus entry mediator (HVEM). In mouse, PVRL1 knockout was reported to attenuate but not abolish HSV infection (Taylor et al. 2007), and PVRL1 was essential for lethal brain infection when inoculated directly (Kopp et al. 2009), but not peripherally. By contrast, TNFRSF14 (HVEM) was not essential (Kopp et al. 2009). The current view is that receptor-bound gD leads to activation of gH/gL, which in turn transforms gB into a fusion-competent state (Campadelli-Fiume et al. 2012a). Virus-induced membrane fusion, however, is only possible if gB is bound by an additional specific gB receptor. Three gB receptors are currently known, including (i) the paired immunoglobulin-like type 2 receptor α (PILRA), (ii) another paired-type receptor with homology to PILRA, myelin-associated glycoprotein (MAG), and (iii) myosin heavy chain 9 (MYH9, non-muscle, also known as NMMHC-IIA). There is also some evidence that HSV-1 entry may require the binding of the gH/gL heterodimer to a cellular receptor (Campadelli-Fiume et al. 2012b; Karasneh and Shukla 2011).

In vitro, HSV-1 infects a diverse spectrum of different human and non-human cells. In vivo, however, infection is much more restricted to epithelial and neuronal cells. Despite extensive characterization of HSV-1 receptors, little is known about their tissue expression patterns in the human brain. In mouse, PVRL1/nectin 1 expression has been reported in limbic regions, frontal association cortex, and olfactory system (e.g., Horvath et al. 2006), but few studies are available in human. Guzman et al. (2006) and Geraghty et al. (1998) reported expression of PVRL1/nectin 1 in a variety of normal and transformed human cells, but the brain distribution was not analyzed. In the most detailed study of human brain, Prandovszky et al. (2008) used immunohistochemistry to inspect fetal human tissues and reported PVRL1 expression in endometrium, cornea, and cortex, with strong staining in pyramidal cells of the Hpc. However, no studies have addressed the expression of HSV-1 receptors other than PVRL1 in human brain. In mice, there are marked changes in receptor expression between the fetal and postnatal periods (Horvath et al. 2006; Prandovszky et al. 2008), but the patterns of HSV-1 receptor expression in adult human brain have not been examined. We therefore used different online transcriptome databases to map the expression patterns of all known HSV-1 gD and gB receptors in adult and developing human brain.

## Methods

### HSV-1 receptor genes

The following HSV-1 receptor genes were used in this study: *PVRL1* (NM_002855), *TNFRSF14* (NM_001297605), *PILRA* (NM_013439), *MYH9* (NM_002473), and *MAG* (NM_001199216). As a positive control gene for the limbic brain, we used the mineralocorticoid receptor *NR3C2* (NM_000901) that is selectively expressed in the Hpc. The housekeeping gene glyceraldehyde-3-phosphate dehydrogenase (*GAPDH*) (NM_001256799) was used as a reference control. Where *GAPDH* data were not available, *GAPDH2/GAPDHS* (NM_014364) was employed instead.

### Extraction of expression profiling data

Expression profiling results were first extracted from the microarray-based Allen Human Brain Atlas (ABA; v.1 March 2013; brainmap.org) of human brain gene expression (Hawrylycz et al. 2012) using the syntax GENE1 OR GENE2. The database is generally based on at least two probes per gene and brain samples from six individuals. The ABA heatmap image was toggled to align all results by region; the image and associated *z* score data were downloaded. To evaluate fold changes in expression levels, the ABA human gene database was interrogated in differential mode for Hpc versus a control brain tissue, cerebellum. For developmental expression profiling, the microarray-based Human Brain Transcriptome database (consulted August 2016; http://hbatlas.org/pages/hbtd) was employed. Expression levels (log_2_ values) at different developmental stages were calculated from the trendline presented for each gene; each point is dependent on at least three independent measurements for a single hybridization probe. Bodywide expression profiles were determined from https://www.ncbi.nlm.nih.gov/geoprofiles, https://www.ebi.ac.uk/gxa/home, and http://biogps.org.

### Data analysis and statistics

Primary ABA *z* scores were imported into OpenOffice (www.openoffice.org) spreadsheets for analysis and graphical plots. Normalized expression profiling results with positive *z* scores were presented as a heatmap with black (*z* = 0, no difference from mean), dark red (*z* = +1, 1 × SD above mean), and bright red (*z* = +2, 2 × SD above mean). For numeric fold change data, Hpc gene expression was compared to cerebellum, and data for 29,509 probes were downloaded as batches of 2000 rows including *P* values and fold changes and searched manually for the listed HSV-1 receptor genes (for details of computations see brainmap.org). For the HBT data, log_2_ values were converted to absolute values before calculation of means and SD; *P* values for Hpc versus neocortex and cerebellum employed the Student *t* test.

## Results

### Expression in adult human brain

**Fig. 1.**
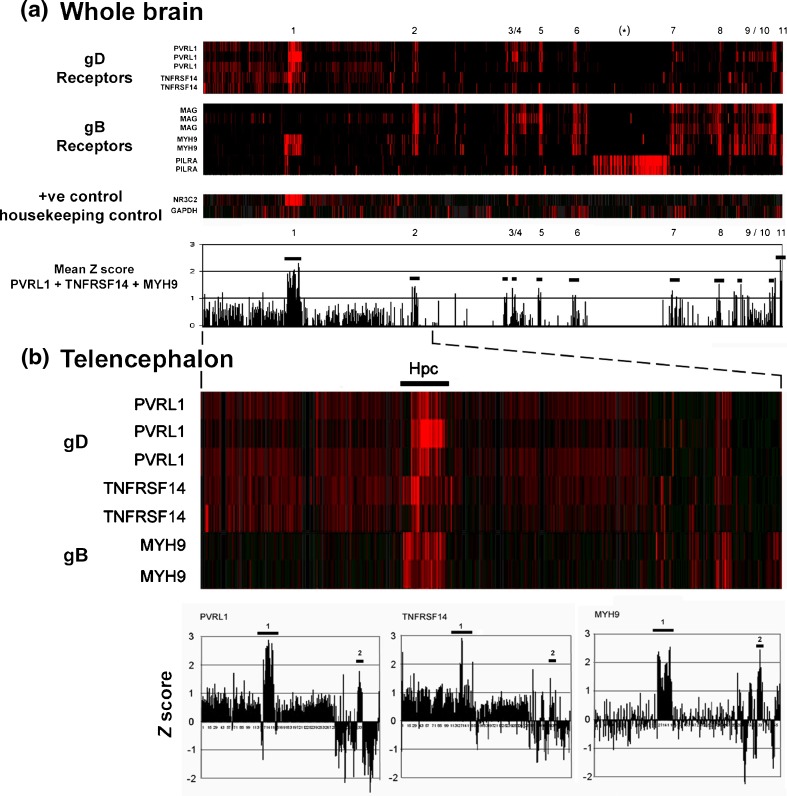
Distribution of HSV-1 gD and gB receptor expression across **a** the whole human brain and **b** telencephalon (data: Allan Brain Atlas). Brain regions (*numbered*) are *1* hippocampus, Hpc; *2* globus pallidus; *3*/*4* subthalamus, lateral and geniculate nuclei; *5* ventral thalamus; *6* red nucleus; *7* dentate/fastigial/globose nuclei; *8* superior olivary complex and trigeminal nuclei; *9*/*10* cuneate/dorsal motor/gracile/vestibular nuclei and corpus callosum; *11* choroid plexus of the lateral ventricle. *Asterisk*, cerebellum, high expression of *PILRA* (gB) but low expression of gD receptors. The positive control for region-specific expression was *NR3C2* (mineralocorticoid receptor); the housekeeping gene control was glyceraldehyde-3-phosphate dehydrogenase (*GAPDH*). The lower panel in **a** gives the plotted mean *z* scores for *PVRL1* + *TNFRSF14* + *MYH9* (negative scores not presented); the lower panel in **b** gives the individual *z* scores for *PVRL1*, *TNFRSF14*, and *MYH9*

Currently, both glycoprotein D (gD) and group glycoprotein B (gB) receptors are known to be essential for HSV-1 viral entry (Table 1). To address their distribution in adult human brain, we extracted the expression profiles as *z* scores (see “Methods” section) from the microarray-based Allen Brain Atlas (ABA) gene expression database of adult human brain (Hawrylycz et al. 2012). Controls were housekeeping gene *GAPDH* and the Hpc-selective gene *NR2C3* (mineralocorticoid receptor). In Fig. 1a, the *z* scores for HSV-1 gD and gB receptors are plotted across the human brain, and Fig. 1b shows their distribution in the telencephalon.

**Table 1.** Known gD and gB receptors for HSV-1

Receptor	Gene/protein symbol	Aliases	Protein Family	Tissue expression^a^
*gD receptors*				
Tumor necrosis factor receptor superfamily, member 14 (herpesvirus entry mediator, HVEM)	TNFRSF14	ATAR, CD270, HVEA, HVEM, LIGHTR, TR2	TNF receptor superfamily	Immune cells, broadly expressed
Poliovirus receptor-like 1 (herpesvirus entry mediator C)	PVRL1	NECTIN1, CD111, CLPED1, ED4, HIgR, HVEC, OFC7, PRR, PRR1, PVRR, SK-12	Immunoglobulin superfamily	Epithelial cells, broadly expressed
*gB receptors*				
Paired immunoglobulin-like type 2 receptor α	PILRA	FDF03	Immunoglobulin superfamily	Gastrointestinal tract, genital tract, lung
Myelin-associated glycoprotein	MAG	GMA, SIGLEC-4A, S-MAG	Myelin protein family	Brain
Myosin heavy chain 9, non-muscle	MYH9	DFNA17, FTNS, EPSTS, NMMHC-IIA	Myosin protein family	Bone marrow, lung, GI tract, genital tract

^a^Bodywide tissue distribution profiles were determined using publicly available databases at https://www.ncbi.nlm.nih.gov/geoprofiles, https://www.ebi.ac.uk/gxa/home, and http://biogps.org

Some brain regions were found to express only one type of receptor. For example, the cerebellum (*) expresses elevated levels of the gB receptor gene *PILRA*, but this was not accompanied by elevated levels of gD receptor gene expression. Two brain regions, however, show a highly significant (*z* = >2.0) enrichment for the expression of both types of HSV-1 receptors—the Hpc (region 1 in Fig. 1) and the choroid plexus (CP, region 11) of the lateral ventricle, which lies in close proximity to the Hpc. These two regions prominently express genes for both gD (*PVRL1* and *TNFRSF14*) and gB (*MYH9*) HSV-1 receptors. By contrast, no enriched expression of *GAPDH* was observed (*z* = 0.0), whereas the positive control, *NR2C3*, was strongly and selectively expressed in Hpc as expected (*z* = 3.1).

In addition to the Hpc and the choroid plexus, several regions (2–10 in Fig. 1) such as the globus pallidus (Gp, 2) display elevated but less-pronounced (*z* ≥ 1.0) expression of both gD and gB receptors, predominantly *PVRL1* and *MAG*.

We also compared HSV receptor profiles between male and female brain. Overall, the patterns were highly similar, with selective Hpc expression in both male and female, and close inspection revealed no major differences, although potential differential receptor expression was noted in two regions: the choroid plexus, where high-level expression was present in male but not female, and the lateral hypothalamic area (anterior region), where expression was elevated in female but not in male (data not presented). However, because the ABA microarray profiles are based on a single female individual, no firm conclusions may be drawn.

To obtain an estimate of the actual level of overexpression of these receptors in Hpc, the ABA gene expression database was interrogated in differential mode in which Hpc was compared against a control brain region, the cerebellum. As shown in Table 2, the positive control *NR3C2* is overexpressed by a factor of 3.25, whereas the housekeeping control showed no overexpression (1.04). The three key HSV-1 receptor genes, *MYH9*, *PVRL1*, and *TNFRSF14*, were 2.07-, 3.06-, and 1.84-fold overexpressed in Hpc, respectively (*P* < 0.001 in all cases) (Table 2).

**Table 2.** Relative transcript levels of HSV-1 receptors in adult human hippocampus versus cerebellum

Database	ABA			HBT	
Gene	Fold change score^a^	Mean fold change	*P* value^a^	Mean fold change^b^	*P* value^b^
Controls					
*NR3C2*	3.33^c^	3.25	8.21E−80	1.85	<0.001
	3.17^c^		1.53E−74		
*GAPDH*	1.04	1.04	ns	nd	na
*GAPDH2*	na	na	na	0.98	ns
HSV receptors					
*MAG*	ne	ne	na	2.90	<0.001
*MYH9*	2.15	2.07	1.92E−44	1.99	<0.001
	1.98		5.01E−57		
*PILRA*	ne	ne	na	ne	na
*PVRL1*	4.04	3.06	1.11E−46	1.40	<0.001
	2.59		5.62E−38		
	2.54		4.55E−38		
*TNFRSF14*	2.49	1.84	4.39E−56	nd	nd
	1.18		5.84E−03		

*ABA* Allan Brain Atlas, *HBT* Human Brain Transcriptome database, *na* not applicable, *nd* no data, *ns* not significant, *ne* not enriched in hippocampus

^a^Fold change overexpression scores were determined by comparing levels of gene expression in Hpc versus a control brain region (cerebellum, Cb); *P* values and fold change scores were computed online at ABA

^b^Fold change overexpression scores are for adult Hpc versus cerebellar cortex and are means of values for age-points 10,000 days (adult) and 30,000 days only (elderly adult); *P* values for Hpc versus cerebellar cortex, Student *t* test

^c^Different entries for each gene correspond to different hybridization probes for the same target (cf Fig. 1)

We also consulted the ABA to compare human versus mouse brain profiles of HSV receptor expression. Although the small size of the mouse brain does not lend itself to accurate interrogation of gene expression patterns in specific substructures (e.g., brainstem nuclei), this analysis confirmed enriched expression of *Myh9* (gB) and *Pvrl1* (gD) in Hpc, in accordance with previous data for *Pvrl1* (“Introduction” section). Mouse data for *Tnfrs14* were not reliable, and for *Pilra*, no overexpression in cerebellum was present (data not presented), in contrast to the situation for human. Overall, we conclude that enriched expression of HSV receptors in adult Hpc is a feature of both mouse and human.

### Developmental profile of HSV-1 receptor expression

There are marked changes in mouse brain expression profiles between gestational and postnatal/adult periods (“Introduction” section). To address the developmental profile of human HSV-1 receptors, we consulted the Human Brain Transcriptome (HBT) database (Johnson et al. 2009; Kang et al. 2011). This also provided independent verification of results generated by ABA. HBT data are for a range of ages, but for a restricted number of brain regions, and to simplify analysis, we focused on five developmental stages as follows: 140 days (mid-gestation), 655 days (1 year postnatal), 2000 days (4.7 years, child), 10,000 days (26.6 years, adult), and 30,000 days (81.4 years, elderly). We also focused on three specific brain regions: Hpc (in which enrichment of HSV receptors in adult brain was indicated, see earlier); neocortex (NCX, another telencephalon region, cf. Fig. 1b); and cerebellar cortex (CB, control for differential overexpression calculations, cf. Table 2). Because the HBT analysis is based on a commercial chip, data for the housekeeping control *GAPDH* were unavailable; *GAPDH2* (also known as *GAPDHS*) was used instead. In addition, no data were available for *TNFRSF14*.

**Fig. 2.**
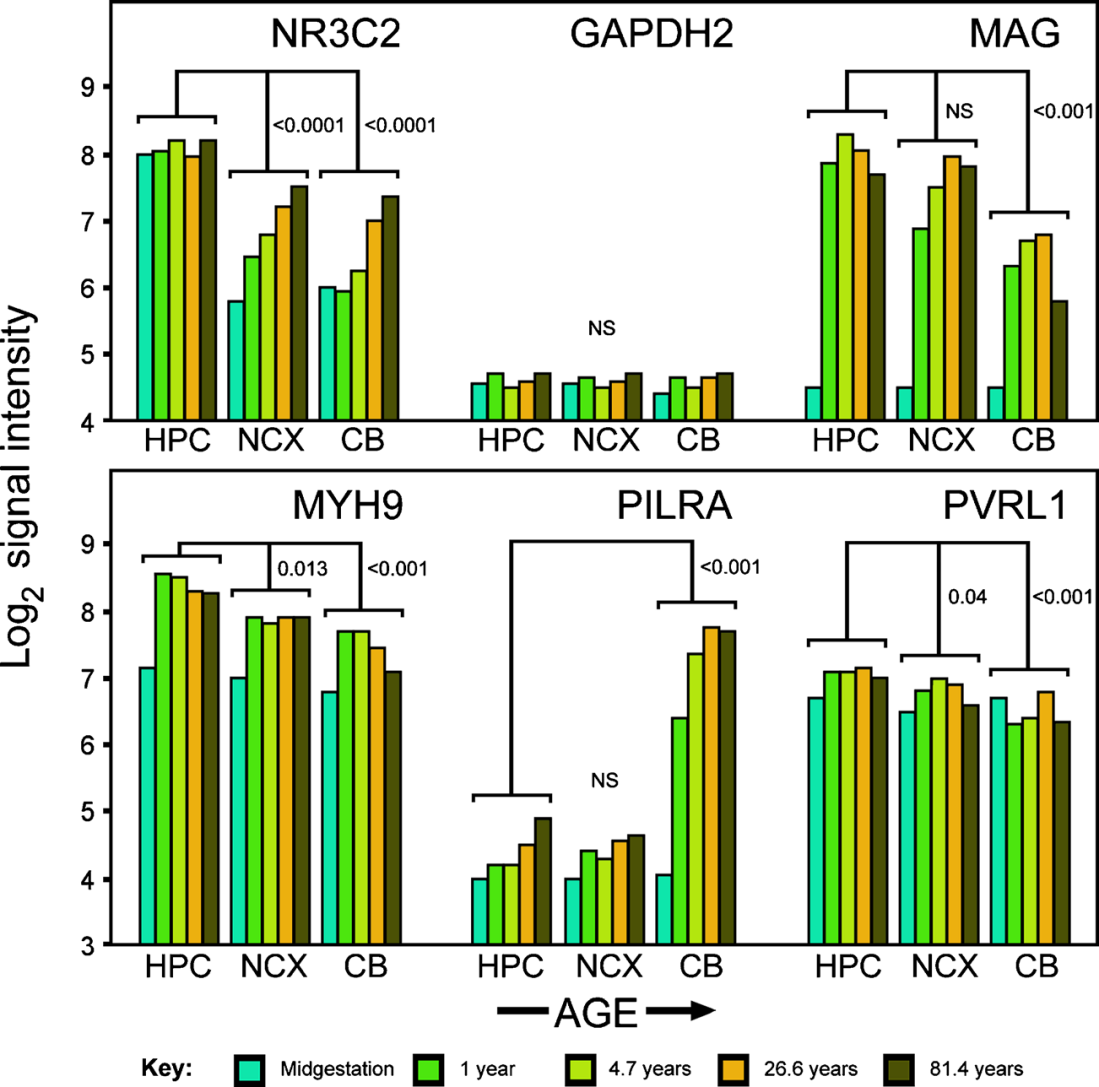
Developmental profiling of HSV-1 receptor expression (log scale) in three different brain regions and across five developmental stages (data: Human Brain Transcriptome). Abbreviations: *CB* cerebellar cortex, *HPC* hippocampus, *NCX* neocortex. No data for *TNFRSF14* were available. *NR3C2* (mineralocorticoid receptor), positive control for hippocampal expression; *GAPDH2*, housekeeping control. *P* values: Student *t* test

The results (Fig. 2) confirmed marked developmental changes in the levels of expression of the HSV-1 receptors analyzed. Overall, expression was much lower during gestation than in postnatal life, with major upregulation taking place between mid-gestation and 1 year of age for *MAG* and *MYH9*. For *MAG*, *MYH9*, and *PVRL1*, overall expression levels averaged across all ages were significantly higher in Hpc than in cerebellum, in agreement with our results obtained from adult human brain (this work) and with a previous report of enriched *PVRL1* expression in Hpc of fetal brain (Prandovszky et al. 2008). Mean fold overexpression levels (Hpc versus cerebellum) across all ages were 2.90, 1.74, and 1.45 for *MAG*, *MYH9*, and *PVRL1*, respectively (values for adult only are given in Table 2); fold overexpression for *NR3C2* (all ages) was 2.72, whereas the housekeeping control showed no enrichment (1.02-fold).

For *PILRA*, in confirmation of Fig. 1 data, adult cerebellar expression levels were significantly higher than in Hpc, but this was only seen later in development, and in mid-gestation levels were uniformly low.

A major discrepancy between ABA and HBT emerged, with high levels of *MAG* expression being seen in postnatal Hpc in the HBT database, whereas these regions were not enriched over mean brain levels in ABA. Although less authoritative than either ABA or HBT, Caracausi and co-workers report no enrichment of *MAG* expression in human Hpc (Caracausi et al. 2016), supporting ABA. However, a question mark remains regarding whether *MAG* is expressed selectively in the Hpc versus other brain regions.

In summary, this analysis confirms enriched expression in Hpc for three HSV-1 receptor genes, *MYH9* (gB receptor), *PVRL1* (gD), and *TNFRSF14* (gD), whereas data for *MAG* (gB) were discordant, but possibly also pointing to enriched expression in Hpc. By contrast, *PILRA1* (gB) was prominently expressed in cerebellum. In all cases, there was a marked increase in expression levels between gestation and postnatal life.

## Discussion

Type 1 herpes simplex virus (HSV-1) is a neurotropic virus that affects select brain regions including particular brainstem nuclei and, most notably, the temporal brain including the Hpc, both in mouse and in human. HSV-1 is unusual among viruses in that it requires the expression of two different receptors on the same target cell, which respectively interact with viral glycoproteins gB and gD (“Introduction” section). We present for the first time a systematic analysis of the distribution of both gB and gD HSV-1 receptors in human brain. Our results confirm and extend previous results, in mouse, that the gene encoding the HSV-1 receptor PVRL1 is selectively expressed in Hpc (Horvath et al. 2006), and we now report that, in adult human, at least three HSV-1 receptors are differentially expressed in Hpc, *MYH9* (gB receptor), *PVRL1* (gD), and *TNFRSF14* (gD), whereas *PILRA* (gB) is most abundantly expressed in human cerebellum. For *MYH9*, *PVRL1*, and *TNFRSF144*, the level of upregulation in Hpc was comparable to that observed for the mineralocorticoid receptor, the archetypical Hpc-specific transcript in mouse (discussed below). *MAG* (gB) expression may also be enriched in Hpc, but there was major discrepancy between the two databases we consulted (ABA and HBT), with high levels of *MAG* expression being seen in postnatal Hpc in the HBT database, whereas this region was not apparently enriched over mean brain levels in ABA. We have no explanation for this result, but possible explanations include the use of different hybridization probes that might detect alternative transcript splicing and/or differential background hybridization, noting that ABA is in general based on two or more probes per gene, whereas HBT relies on single probes.

Although the different human brains sampled in ABA gave slightly different expression patterns for the same hybridization target, in this study the overall profile was largely conserved (Fig. 1); technical issues with tissue preparation combined with genetic and environmental effects may underlie this variability.

In addition, patterns were substantially conserved between male and female brain, with selective HSV receptor expression in Hpc in both male and female. There were potentially some minor differences (“Results” section); however, the ABA microarray database is based on a single female individual, and studies on a greater diversity of individuals will be necessary to address possible male/female differences in HSV receptor expression.

Both databases confirm that *PILRA* (gB receptor) is prominently expressed in cerebellum; however, there was no parallel enrichment of gD receptors: lack of gD receptors could explain the observation, in mouse, that inoculation of HSV into cerebellum did not give rise to severe disease (McFarland and Hotchin 1987).

Significant gB/gD receptor expression was also observed (ABA) in adult globus pallidus (Gp), trigeminal nuclei, brainstem, and red nucleus. This is of interest because HSV-1 brain infection can sometimes selectively involve the Gp (Hu et al. 2003). The trigeminal nuclei are also of note because they are connected to the trigeminal ganglia where HSV-1 latently persists (Baringer and Swoveland 1973; Croen et al. 1987; Furuta et al. 1992; Theil et al. 2001), and selective involvement of the brainstem in some cases of HSV-1 encephalitis has been reported (Livorsi et al. 2010). Finally, the presence of receptors in the red nucleus is notable because this region is functionally connected with the Hpc (Dypvik and Bland 2004; Nioche et al. 2009). The relevance of other brain regions highlighted in Fig. 1 to the pathobiology of HSV-1 infection is not known and warrants further investigation.

The finding that HSV-1 receptors are expressed at only low levels in gestation, but rise markedly during the postnatal period, is notable because the fetal human brain is not generally subject to infection with HSV-1, in contrast to other viruses (e.g., cytomegalovirus, rubella, Zika virus) that can establish damaging brain infection during gestation (Driggers et al. 2016; Guillemette-Artur et al. 2016; McCarthy et al. 2011; Naeye and Blanc 1965). Although exposed to HSV-1 in utero of HSV-1-positive mothers, overt pathology appears to be generally limited to postnatal ages. Our results suggest that the fetal human brain may be partially refractory to HSV-1 infection owing to low levels of receptor expression.

This study has some limitations. First, the data presented address transcript levels rather than protein expression. Brain proteomic analysis demonstrates expression of PVRL1 in adult human Hpc (Hondius et al. 2016), but other brain regions were not studied; comprehensive proteomics of human brain (an emerging project at www.hupo.org/human-brain-proteome-project) will be necessary to confirm the regional distribution of HSV-1 receptors. Second, cell-surface receptors are not the only determinants of virus tropism, and multiple levels of control on virus entry, genome transcription, and translation govern the outcome of infection. Additional restriction factors include routing factors such as αVβ3 integrin (Campadelli-Fiume et al. 2012a), which affect virus internalization, and also proteins such as B5 that influence HSV-1 translation (Cheshenko et al. 2010; Perez et al. 2005). However, without expression of receptors for both gD and gB, effective virus entry cannot take place. Third, much of our analysis addresses the normalized *z* score or fold-change values instead of absolute expression levels. However, this is not necessarily misleading. To illustrate, mineralocorticosteroids are known to selectively target the Hpc, and, for the mineralocorticoid receptor *NR3C2*, the top-ranked gene for selective expression in mouse Hpc (Lein et al. 2007), both the *z* and fold change scores were comparable to those generated by both gD and gB receptors (Fig. 1 and Table 2).

Further, because HSV-1 entry requires the simultaneous expression of at least two different receptors, conjoint overrepresentation of both receptor types in a single tissue even by a small amount may be anticipated to synergistically and disproportionately influence the statistical likelihood of encountering both types of receptor on the same cell, further amplified during serial transfer between cells, and thus favor selective tissue tropism. For example, if there is only a 2-fold excess of receptors for gB (MYH9) and gD (PVRL1 plus TNFRSF14), the differential efficiency of primary infection could rise 8×, and to 64× for a further round of infection.

In conclusion, the selective virus receptor gene expression profiles reported here may contribute to the tropism of HSV-1 for particular postnatal brain regions in human, centrally including the temporal brain and Hpc.
